# A Systematic Review and Meta-Analysis of Exercise Beneficial for Locomotion in Community-Dwelling Elderly People with Sarcopenia

**DOI:** 10.3390/jfmk8030092

**Published:** 2023-06-29

**Authors:** Seunghyeok Song, Gushik Kim, Hyunjoong Kim

**Affiliations:** 1 Korea Pediatric Integrative Manual Therapy Association, 302 Gwanggyojungang-ro, Yongin 16943, Republic of Korea; 2 Gyeonggi Branch, Korea Physical Therapy Association, 1030 Gyeongsu-daero, Suwon 16203, Republic of Korea; 3 Neuromusculoskeletal Science Laboratory, 306 Jangsin-ro, Gwangju 62287, Republic of Korea

**Keywords:** aged, sarcopenia, locomotion, muscle strength, exercise, gait

## Abstract

Sarcopenia, in addition to aging and reduced physical activity, is a progressive skeletal muscle disorder that causes the loss of muscle mass and strength. The most prominent functional change is mobility, which contributes to a decrease in the quality of life. Therefore, we aimed to perform qualitative and quantitative analyses by synthesizing randomized controlled trials (RCTs) that evaluated exercises that affected locomotion in patients with sarcopenia. The RCTs were retrieved in April 2023 from three international electronic databases (Embase, MEDLINE, and PubMed). RCTs published after 2013 were compared with a control group that did not include exercise. Qualitative and quantitative analyses were performed on the identified studies using RevMan 5.4 and risk of bias assessment provided by Cochrane. RCTs involving 594 patients with sarcopenia were included in this study. The analysis model was synthesized as a random effects model, and the standard mean difference (SMD) was used as the effect measure. Exercise interventions were found to not change muscle mass in individuals with sarcopenia (SMD = 0.04; 95% CI: −0.15 to 0.22). However, they had positive effects on lower extremity muscle strength (SMD = 0.34; 95% CI: 0.02 to 0.66) and walking speed (SMD = 0.42; 95% CI: 0.11 to 0.72). For community-dwelling elderly people with sarcopenia, exercise intervention did not lead to an increase in reduced muscle mass, but it brought positive improvements in lower extremity strength and gait speed to improve locomotion.

## 1. Introduction

Sarcopenia is a progressive skeletal muscle disorder characterized by the accelerated loss of muscle mass and subsequent deterioration in muscle function [1]. With advancing age, there is a significant decline in muscle mass, which is estimated to be around 40% from the age of 25 years to after the age of 80 years [2]. This decline is further exacerbated by reductions in physical activity levels which accelerate the loss of muscle mass and contribute to declines in physical function [3]. The cumulative effects of aging, sedentary behavior, and ongoing muscle loss have profound implications for the quality of life and increased mortality rates in individuals with sarcopenia [4,5].

Understanding the effects of acute or chronic exercise on sarcopenia necessitates consideration of age-related variations in endocrine factors [1]. Recent systematic reviews and meta-analyses have highlighted that group-based and supervised resistance training, along with multicomponent exercise, are particularly effective in enhancing muscle function and performance [6]. Nevertheless, despite these findings, there remains a paucity of consistent information regarding the effectiveness of exercise interventions in managing sarcopenia.

Health-related quality of life is a critical determinant in the elderly population, and the reduction in mobility associated with sarcopenia is an inevitable consequence [7]. Studies exploring the association between quality of life and mobility have consistently reported that mobility limitations among older adults further contribute to the deterioration of their overall well-being [8]. Reduced locomotion significantly impacts an individual’s ability to engage in daily activities, making mobility a crucial factor influencing the lifestyles of individuals with sarcopenia.

To address knowledge gaps, this systematic review and meta-analysis aimed to comprehensively analyze randomized controlled trials (RCTs) and investigate the impacts of exercise interventions on locomotion in individuals with sarcopenia. Both qualitative and quantitative analyses were conducted, facilitating a robust comparison and evaluation of exercise effects. By synthesizing the existing evidence, this study aims to provide a comprehensive understanding of the effects of exercise on locomotion in individuals with sarcopenia and contribute to the development of effective exercise interventions targeting this population.

## 2. Materials and Methods

### 2.1. Study Design

Our study conducted a comprehensive systematic review and meta-analysis, focusing on the impact of exercise on locomotion in community-dwelling elderly individuals with sarcopenia. Adhering to the guidelines of the Preferred Reporting Items for Systematic Reviews and Meta-Analysis (PRISMA), our study was meticulously prepared. Furthermore, the study protocol was registered with the international prospective register of systematic reviews (PROSPERO) under the registration number CRD42023391773, ensuring transparency and adherence to the highest research standards.

### 2.2. Search Strategy and Selection of Studies

The search strategy and study selection process followed the guidelines of PICOSD (participants, interventions, comparisons, outcomes, and study design) to establish the inclusion and exclusion criteria.

#### 2.2.1. Inclusion Criteria

The inclusion criteria for this review were determined based on the PICOSD framework. The participants consisted of community-dwelling elderly individuals diagnosed with sarcopenia. The interventions involved a range of exercises and training programs incorporating physical activity. Comparisons were made with usual care or control groups. The outcome measures focused exclusively on variables (gait speed, walking speed, locomotion, muscle activity, muscle strength, and lower extremity strength) related to locomotion. The study design specifically targeted randomized controlled trials (RCTs) sourced from the database.

#### 2.2.2. Exclusion Criteria

Th exclusion criteria for this review included studies not written in English, studies involving physical activity in a control group, and studies involving older adults with musculoskeletal or neurological disorders. In addition, when considering the qualitative factors of study design and the trends of intervention, studies not published within the last 10 years were excluded (studies published before 2013).

#### 2.2.3. Literature Search Strategy

This review was conducted through a systematic search for relevant studies published before April 2023, which coincided with the PROSPERO registration period. The search was performed by a team of experienced researchers who are well versed in meta-analyses. They employed a comprehensive set of keywords to capture the relevant literature, including terms such as elderly, older adults, aged, sarcopenia, gait speed, walking speed, locomotion, muscle activity, muscle strength, lower extremity strength, and RCTs. The search strategy utilized a logical expression, combining these keywords using the Boolean operator “AND”, to ensure the inclusion of studies that focused on the intersection of these concepts. The final search expression used was ((elderly OR older adults OR aged) AND (sarcopenia) AND (gait speed OR walking speed OR locomotion OR muscle activity OR muscle strength OR lower extremity strength) AND (randomized controlled trial)) (Table 1).

The international electronic databases used for the search were the Excerpta Medica (Embase), Medical Literature Analysis and Retrieval System Online (MEDLINE), and PubMed databases.

#### 2.2.4. Study Selection and Data Extraction

To ensure data accuracy, the process of extracting studies from the electronic databases involved removing any duplicate papers using Microsoft Excel (Microsoft, Redmond, WA, USA). Following the PRISMA flow diagram, an initial screening was conducted based on the titles and abstracts of the identified papers. Subsequently, the full texts of the selected papers were thoroughly reviewed, considering pre-established eligibility criteria. The included papers were then categorized based on their characteristics, and both qualitative and quantitative analyses were performed to extract meaningful insights.

#### 2.2.5. Quality Assessment

To assess the quality of the included randomized controlled trials (RCTs), the Cochrane Risk of Bias (RoB) tool was employed [9]. This tool comprises seven items that evaluate various aspects of study quality, including random sequence generation, allocation concealment, blinding participants and personnel, blinding the outcome assessment, incomplete outcome data, selective reporting, and other potential biases. Each item was assessed to determine the presence of a low bias (+), uncertain bias (?), or higher bias (−). The quality assessment was conducted independently by each researcher, and any discrepancies were resolved through a consensus process to reach a final decision.

### 2.3. Strategy for Data Synthesis

The selected papers were synthesized and analyzed using RevMan 5.4, a software developed by The Cochrane Collaboration in Oxford, England. When three or more papers reported the same variables, they were included in the quantitative and meta-analyses. To account for heterogeneity among the included studies, a random effects model was employed for the analysis. The effect sizes were assessed using the standard mean difference (SMD).

Heterogeneity between studies was assessed using the Chi-square test and the I2 test. The interpretation of the I2 results was as follows: values greater than 75% indicated a high level of heterogeneity, while values below 40% suggested a low level of heterogeneity [10]. Furthermore, if more than 10 papers were included in the analysis, a potential publication bias was evaluated using a funnel plot [11], which provided a visual assessment. The statistical analyses were performed using Egger’s test with statistical software (SPSS Version 29.0; IBM Corp., Armonk, NY, USA).

## 3. Results

### 3.1. Literature Search and Characteristics of the Included Randomized Clinical Trials

**Figure 1:**
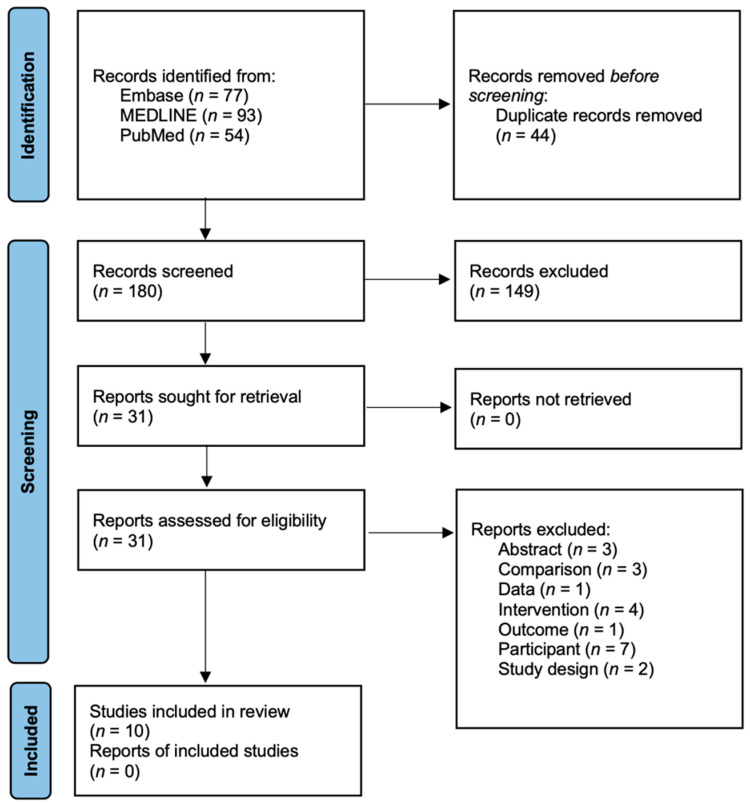
Preferred Reporting Items for Systematic Reviews and Meta-Analysis (PRISMA) flow diagram.

A comprehensive search of three international databases using pre-defined index terms yielded a total of 224 papers. After removing 44 duplicate papers, the titles of the remaining 180 papers were transferred to Excel for further analysis. Through the screening process, as depicted in Figure 1, a total of 149 papers were excluded based on their titles and abstracts. Subsequently, the full texts of the remaining 31 papers were carefully reviewed according to the predetermined eligibility criteria, resulting in the exclusion of an additional 21 papers. Finally, ten papers were deemed eligible and included in our systematic review and meta-analysis [12,13,14,15,16,17,18,19,20,21].

### 3.2. Risk of Bias Assessment

In our systematic review, a rigorous assessment of the risk of bias was conducted to ensure the reliability and validity of the included studies. The assessment results demonstrated a high level of agreement (100%) among the researchers involved in the evaluation process. The risk of bias assessment encompassed various aspects of study design and conduct, including random sequence generation, allocation concealment, blinding participants and personnel, blinding the outcome assessment, incomplete outcome data, selective reporting, and other biases.

**Figure 2:**
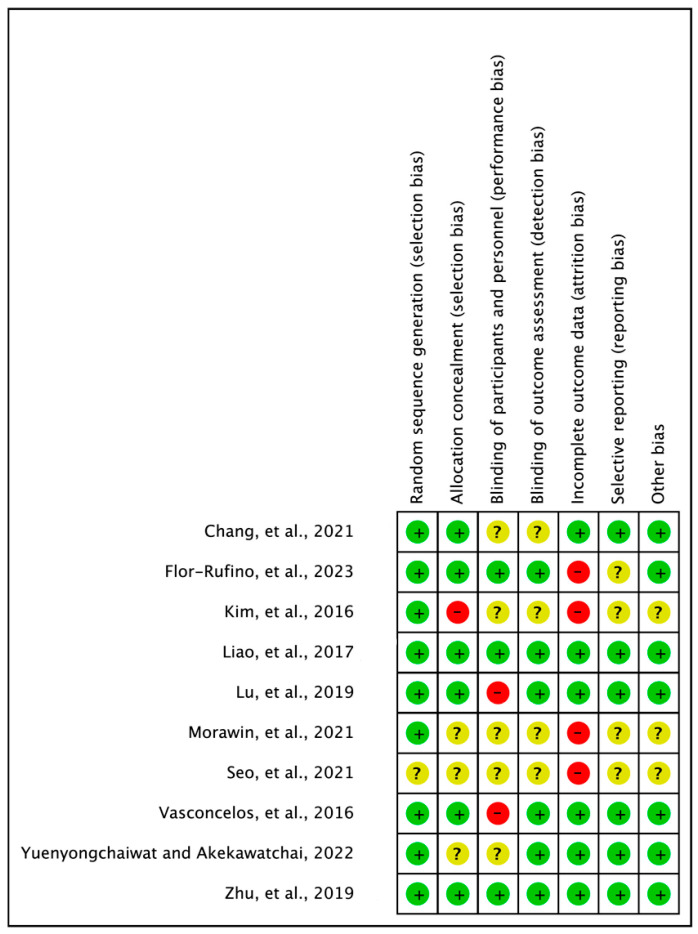
Risk of bias summary: review of authors’ judgements about each risk of bias item for each included study. Chang et al., 2021 [15], Flor-Rufino et al., 2023 [21], Kim et al., 2016 [20], Liao et al., 2017 [18], Lu et al., 2019 [12], Morawin et al., 2021 [14], Seo et al., 2021 [16], Vasconcelos et al., 2016 [17], Yuenyongchaiwat and Akekawatchai, 2022 [13], Zhu et al., 2019 [19].

The findings of the risk of bias assessment revealed the following distribution of ratings for each criterion: random sequence generation (low: 9; uncertain: 1), allocation concealment (low: 6; uncertain: 3; high: 1), blinding participants and personnel (low: 3; uncertain: 5; high: 2), blinding the outcome assessment (low: 6; uncertain: 4; high: 0), incomplete outcome data (low: 6; uncertain: 0; high: 4), selective reporting (low: 6; uncertain: 4; high: 0), and other biases (low: 7; uncertain: 3; high: 0). This comprehensive assessment provides insight into the methodological quality and potential biases that may have influenced the findings of the included studies (Figure 2).

It is important to note that the assessment of uncertainty in other biases was based on a careful consideration of various factors, including baseline characteristics, sample size calculations, and adherence to pre-registration protocols, all of which were informed by prior studies [22].

### 3.3. Exercise for Individuals with Sarcopenia

In our systematic review and meta-analysis, a total of 594 community-dwelling elderly individuals with sarcopenia participated across the ten selected randomized controlled trials (RCTs). These trials encompassed a diverse range of interventions targeting the improvement of locomotion in this population. The primary intervention modality utilized was resistance exercises, which were supplemented with additional interventions such as nutritional interventions, comprehensive training programs, home exercise programs, and Tai Chi training.

The durations of the interventions varied among the studies, ranging from three months to six months, allowing for a substantial period of engagement and potential impact on the outcomes of interest. The primary outcome variables assessed in these studies included measurements of muscle mass, lower extremity strength, and gait speed. These variables were chosen as they directly reflect the functional aspects of locomotion and are commonly used to evaluate the effectiveness of interventions in this context.

It is worth noting that while the specific measurement methods employed in the included studies may have differed slightly, all studies captured relevant variables related to locomotion, ensuring consistency and comparability across the synthesized data. A comprehensive overview of the included studies and their respective outcome measures can be found in Table 2, providing a comprehensive summary of the data sources utilized in our analysis.

### 3.4. Effectiveness of Exercise on Muscle Mass

**Figure 3:**
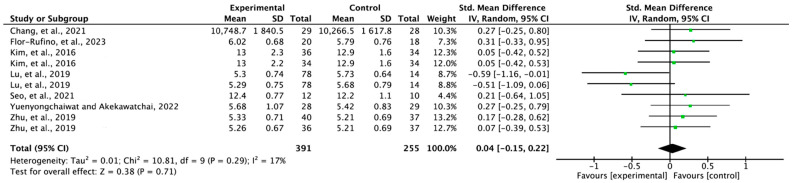
Forest plot of the effect of exercise on muscle mass. Chang et al., 2021 [15]; Flor-Rufino et al., 2023 [21]; Kim et al., 2016 (a) [20], exercise plus nutrition; Kim et al., 2016 (b) [20], physical comprehensive training program; Lu et al., 2019 (a) [12], complex intervention for 3 months; Lu et al., 2019 (b) [12], complex intervention for 6 months; Seo et al., 2021 [16]; Yuenyongchaiwat and Akekawatchai, 2022 [13]; Zhu et al., 2019 (a) [19], exercise alone; Zhu et al., 2019 (b) [19], combined exercise program and nutrition supplement.

For muscle mass, seven papers (*n* = 483) were selected from the ten enrolled papers. The experimental groups that included exercise did not show significant changes in muscle mass compared with the control groups (SMD = 0.04; 95% confidence interval (CI): −0.15, 0.22; heterogeneity (χ^2^ = 10.81; df = 9, I^2^ = 17%), and overall effect (Z = 0.38; *p* = 0.71)). However, the means of the experimental groups were larger in six papers (Figure 3).

### 3.5. Effectiveness of Exercise on Lower Extremity Strength

**Figure 4:**
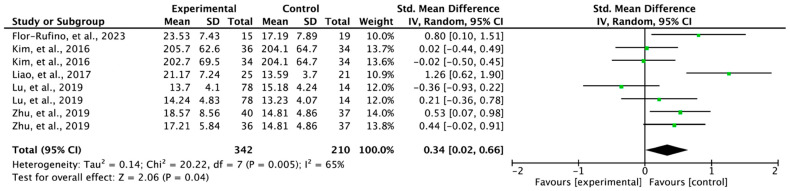
Forest plot of the effect of exercise on lower extremity strength. Flor-Rufino et al., 2023 [21]; Kim et al., 2016 (a) [20], exercise plus nutrition; Kim et al., 2016 (b) [20], physical comprehensive training program; Liao et al., 2017 [18]; Lu et al., 2019 (a) [12], complex intervention for 3 months; Lu et al., 2019 (b) [12], complex intervention for 6 months; Zhu et al., 2019 (a) [19], exercise alone; Zhu et al., 2019 (b) [19], combined exercise program and nutrition supplement.

For lower extremity strength, five papers (*n* = 389) were selected from the ten registered papers. The experimental groups including exercise showed significant changes in lower extremity strength compared to the control groups (SMD = 0.34; 95% CI: 0.02 to 0.66; heterogeneity (χ^2^ = 20.22, df = 7, I^2^ = 65%); and overall effect (Z = 2.06, *p* = 0.04)) (Figure 4).

### 3.6. Effectiveness of Exercise on Gait Speed

**Figure 5:**
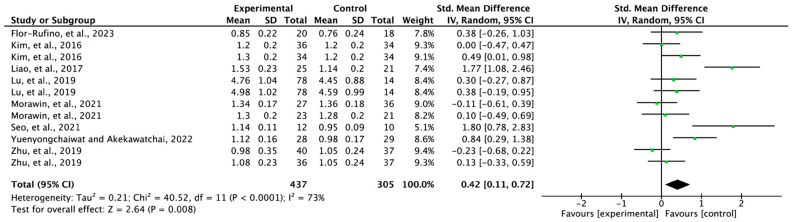
Forest plot of the effect of exercise on gait speed. Flor-Rufino et al., 2023 [21]; Kim et al., 2016 (a) [20], exercise plus nutrition; Kim et al., 2016 (b) [20], physical comprehensive training program; Liao et al., 2017 [18]; Lu et al., 2019 (a) [12], complex intervention for 3 months; Lu et al., 2019 (b) [12], complex intervention for 6 months; Morawin et al., 2021 (a) [14], Tai-Chi training for 4 months; Morawin et al., 2021 (b) [14], Tai-Chi training for 6 months; Seo et al., 2021 [16]; Yuenyongchaiwat and Akekawatchai, 2022 [13]; Zhu et al., 2019 (a) [19], exercise alone; Zhu et al., 2019 (b) [19], combined exercise program and nutrition supplement. Flor-Rufino et al., 2023 [21], Liao et al., 2017 [18], Seo et al., 2021 [16], Yuenyongchaiwat and Akekawatchai, 2022 [13].

For gait speed, eight papers (*n* = 579) were selected from the ten registered papers. The experimental group including exercise showed a significant change in gait speed compared to the control group (SMD = 0.42; 95% CI: 0.11 to 0.72; heterogeneity (χ^2^ = 40.52, df = 11, I^2^ = 65%); and overall effect (Z = 2.64, *p* = 0.008)) (Figure 5).

### 3.7. Publication Bias

**Figure 6:**
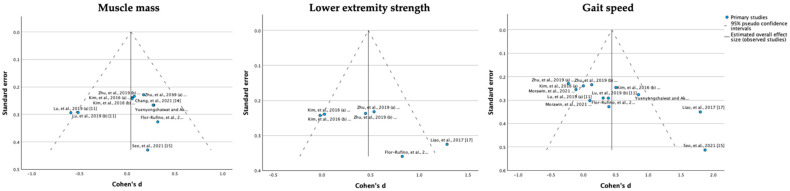
Funnel plot of comparison: muscle mass, lower extremity strength, and gait speed. Chang et al., 2021 [15], Flor-Rufino et al., 2023 [21], Kim et al., 2016 [20], Liao et al., 2017 [18], Lu et al., 2019 [12], Morawin et al., 2021 [14], Seo et al., 2021 [16], Vasconcelos et al., 2016 [17], Yuenyongchaiwat and Akekawatchai, 2022 [13], Zhu et al., 2019 [19].

The assessment of publication bias is shown as a funnel plot in Figure 6. With respect to visual symmetry, two studies deviated for gait speed, while the other outcomes showed relative symmetry. The results of Egger’s test showed a publication bias for gait speed (*p* = 0.002).

## 4. Discussion

Our systematic review and meta-analysis performed qualitative and quantitative analyses and syntheses of RCTs in which community-dwelling elderly individuals with sarcopenia participated in exercise interventions. The purpose of this review was to investigate the effect of exercise on locomotion in individuals with sarcopenia.

### 4.1. Muscle Mass in Individuals with Sarcopenia

The results of the synthesized meta-analysis showed that exercise interventions did not change the muscle mass of individuals with sarcopenia (SMD = 0.04, 95% CI: −0.15 to 0.22) (Figure 3). The therapeutic intensities of the studies that reported changes in muscle mass were as follows, and no studies that produced significant changes were found. Tai chi exercise for 4 to 6 months [14]; high-intensity resistance training for 6 months [21]; exercise plus nutrition and physical comprehensive training for 3 months [20]; complex intervention for 3 to 6 months [12]; resistance training for 16 weeks [16]; exercise and combined exercise program plus nutrition supplement for 12 weeks [19]. Similar to these results, a systematic review and meta-analysis of exercise programs for individuals with sarcopenia did not find any significant results for muscle mass [23]. On the other hand, a meta-analysis based on nutritional interventions reported a significant difference in muscle mass improvement [24].

### 4.2. Lower Extremity Strength in Individuals with Sarcopenia

The results of the synthesized meta-analysis showed that exercise interventions did change the lower extremity strength of individuals with sarcopenia (SMD = 0.34; 95% CI: 0.02 to 0.66) (Figure 4). The interpretation of the effect size showed a small effect (SMD < 0.4) for lower extremity strength. Among the studies that reported changes in lower extremity strength, the greatest effects were found for studies that performed high-intensity resistance training for 6 months [21] and elastic resistance exercise for 12 weeks [18]. The smallest effects were found for a 12-week combined exercise program plus nutrition supplement [19] and 3-month physical comprehensive training studies [20]. These results were reported to be significantly improved in resistance training and mixed training in a previously reported meta-analysis comparing each type of exercise [25].

### 4.3. Gait Speed in Individuals with Sarcopenia

The results of the synthesized meta-analysis showed that exercise interventions did change the gait speed of individuals with sarcopenia (SMD = 0.42; 95% CI: 0.11 to 0.72) (Figure 5). The interpretation of the effect size showed a moderate effect (0.4 ≤ SMD ≤ 0.7) on gait speed. Among the studies reporting changes in gait, large effects was found for studies that performed elastic resistance exercise for 12 weeks [18], resistance exercise for 16 weeks [13], and a walking-based home program for 12 weeks [13]. As with lower extremity strength in a previously reported meta-analysis [25], significant improvements in gait speed were found for resistance exercise and mixed exercise.

### 4.4. Exercise Intervention for Locomotion in Sarcopenic Individuals

Taken together, the results of the synthesized meta-analysis showed that exercise interventions did not change the muscle mass of individuals with sarcopenia (SMD = 0.04, 95% CI: −0.15 to 0.22). However, they had positive effects on lower extremity strength (SMD = 0.34; 95% CI: 0.02 to 0.66) and gait speed (SMD = 0.42; 95% CI: 0.11 to 0.72). Considering that the papers extracted in this review were all RCTs in which exercise interventions were performed for more than three months, the results seem to be relatively poor. In a systematic review and meta-analysis reported in 2018, exercise interventions lasted less than 12 weeks and were effective in improving muscle strength, balance, and mass [26]. However, in contrast to the significant improvement in lower extremity strength and gait speed, excluding muscle mass, there was no significant difference in any of the locomotion variables (muscle mass, lower extremity strength, and gait speed) in this review. In general, 12 weeks was considered an appropriate period for muscle hypertrophy, but specifically considering sarcopenia, 24 weeks was reported to be appropriate [27]. A previous review also set a limitation on the intervention period [26]. Therefore, rather than recommending an exercise intervention appropriate for the muscle mass of individuals with sarcopenia, it is necessary to highlight a preventive method based on exercise habits after middle age [28].

We included interventions involving exercises that affected locomotion in sarcopenia which were not reviewed in order to determine the effect of exercise alone. According to a network meta-analysis comparing nutrition and combined exercise in individuals with sarcopenia [29], there was no difference in muscle mass between the interventions. In addition, for the other variables, no significant differences were found between the group receiving nutritional intervention and the group receiving exercise alone. These results were consistent with our results; furthermore, among the synthesized studies [15,19,20], there was no remarkable effect when the nutritional intervention was combined.

In summary, similar results were presented in previous meta-analyses [26,29], which showed that there were no significant changes in muscle mass. In contrast, positive changes through the use of exercise in all functional variables such as lower extremity strength and gait speed were confirmed. The term sarcopenia indicates only muscle mass; however, its meaning has been extended to muscle function [25]. According to epidemiological studies [30,31], individuals with sarcopenia show a two-to five-fold increase in muscle mass loss. To restore reduced muscle mass, exercise intervention is essential; however, the synthesized results did not show any remarkable positive changes and only showed improvements in muscle function. This can be explained by changes in neurological adaptations such as motor unit recruitment and an increase in the discharge rate [32,33,34,35,36].

Based on these results and mechanisms, we found that exercise interventions for sarcopenia are effective therapeutic options that can delay the loss of muscle mass. In addition, sarcopenia has been reported to increase the risk of death significantly because of the relationship between sarcopenia and mortality [1,37,38,39,40]. Thus, it could be said that regular exercise intervention for more than three months through an early diagnosis is an excellent preventive method for sarcopenia. This review emphasized the relationship between sarcopenia and locomotion. First, a correlation between sarcopenia and depression has been reported [41,42,43,44], and it has been reported that gait patterns can contribute significantly to depression and both are both closely related [42,45,46]. Based on these results, the moderate effect of exercise intervention on gait speed, which could represent the overall gait ability, suggests that it could bring about a positive improvement in depression. In this systematic review and meta-analysis, exercise was found to have beneficial effects on locomotion in community-dwelling older adults with sarcopenia.

### 4.5. Strengths and Limitations

This review had strengths and limitations. In terms of its strengths, it was possible to synthesize and analyze conflicting results from previously reported systematic reviews and meta-analyses [26,29,47,48]. In addition, locomotion was analyzed in relation to the daily life and quality of life of elderly people with sarcopenia. The previous studies and the results of our analysis after synthesis can be summarized into four highlights as follows. First, exercise combined with nutritional intervention was not as effective as exercise alone; second, regarding the exercise period, exercise interventions of three months or longer showed a larger effect size; third, exercise interventions did not significantly improve muscle mass; and fourth, exercise interventions improved muscle function by increasing neurological adaptation to the remaining muscles. However, the limitations were that the results may be biased as we were limited to studies published in English and within 10 years; in terms of the exercise type, the same types of exercise intervention were not analyzed and not selected; papers with different exercise durations were synthesized together; and the most effective gait speed showed the highest heterogeneity.

## 5. Conclusions

Exercise interventions in community-dwelling elderly individuals with sarcopenia did not result in a significant increase in muscle mass, but they did yield positive improvements in lower extremity strength and gait speed, thereby enhancing locomotion.

## Figures and Tables

**Table 1:** Identify keywords.

Participants	Outcomes	Study Design
Elderly OR older adults OR aged	Gait speed OR walking speed OR locomotion OR muscle activity OR muscle strength OR lower extremity strength	Randomized controlled trial
Sarcopenia		

**Table 2:** Characteristics of synthesized clinical trials.

Study	Age, Sample Size	Time Points of Measurement	Intervention Therapeutic Intensity	Outcomes: Unit
Chang et al., 2021 [15]	74.3 ± 5.8, EG = 29; 75.7 ± 5.9, CG = 28	12 weeks	Exercise and nutritional intervention: exercise twice a week, nutritional interventions provided daily (two sticks of branched-chain amino acids daily and two tablets of calcium and vitamin D3 supplement daily). Control = Home exercise program.	Muscle mass: g
Flor-Rufino et al., 2023 [21]	79.9 ± 7.2, EG = 20; 79.6 ± 7.7, CG = 18	6 months	High-intensity resistance training: 6 exercise programs of 65 min per session twice a week for a total of 6 months. Control = Telephone follow-up to assess general health.	Muscle mass: kg/m^2^ LE strength: kg Gait speed: m/s
Kim et al., 2016 [20]	80.9 ± 4.2, EG 1 = 36; 81.4 ± 4.3, EG 2 = 34; 81.1 ± 5.1, CG = 34	3 months	EG 1 = Exercise plus nutrition EG 2 = Physical comprehensive training program; each exercise was conducted for 60 min twice a week for a total of 3 months. Control = Health education.	Muscle mass: kg LE strength: N Gait speed: m/s
Liao et al., 2017 [18]	66.4 ± 4.5, EG = 25; 68.4 ± 5.9, CG = 21	12 weeks	Elastic resistance exercises performed three times a week for a total of 12 weeks; each workout session included a 10 min general warm-up, resistance training (35–40 min), and finally a cool-down routine. Control = No exercise intervention provided.	LE strength: N Gait speed: m/s
Lu et al., 2019 [12]	69.8 ± 4.3, EG = 33; 71.0 ± 6.7, CG = 33	3 months 6 months	Complex intervention: physical exercise, nutritional enhancement, cognitive training, and integrative interventions or standard care for 6 months Control = Standard care.	Muscle mass: kg/m^2^ LE strength: kg Gait speed: s
Morawin et al., 2021 [14]	Mean range: 69.8 to 73.6, EG = 27 CG = 36	4 months 6 months	Tai-Chi training: exercises were performed twice a week, and 2–4 exercises were added monthly. Control = Health education.	Gait speed: m/s
Seo et al., 2021 [16]	70.3 ± 5.38, EG = 12; 72.9 ± 4.75, CG = 10	16 weeks	Resistance training: a total of 48 sessions were provided three times per week. Each training session included a 5 min warm-up, 50 min resistance exercise, and a 5 min cool-down. Control = No exercise intervention provided.	Muscle mass: kg Gait speed: m/s
Vasconcelos et al., 2016 [17]	72 ± 4.6, EG = 14; 72 ± 3.6, CG = 14	10 weeks	Resistance exercise: 10-week resistance exercise program for 1 h twice per week (designed to improve lower extremity strength, power, and endurance through open and closed kinetic chain exercises) Control = Monitored by therapists once per week by phone for a 10-week period.	Gait speed: m/s
Yuenyongchaiwat and Akekawatchai, 2022 [13]	69.23 ± 6.71, EG = 28; 71.93 ± 5.19, CG = 29	12 weeks	Walking-based home program: the intervention program increased PA by encouraging walking, ≥7500 steps daily, for 5 days/week for a total of 12 weeks. Control = Routine daily activities.	Muscle mass: kg/m^2^ Gait speed: m/s
Zhu et al., 2019 [19]	74.5 ± 7.1, EG 1 = 40; 74.8 ± 6.9, EG 2 = 36; 72.2 ± 6.6, CG = 37	12 weeks	EG 1 = Exercise: group exercise and one home exercise twice a week for 12 weeks. EG 2 = Combined exercise program plus nutrition supplement: the nutrition supplement consisted of two sachets of Ensure NutriVigor daily from baseline to 12 weeks. Control = Waitlist.	Muscle mass: kg/m^2^ LE strength: kg Gait speed: m/s

CG—control group; EG—experimental group; LE—lower extremity.

## Data Availability

Not applicable.
